# Identification and Evaluation of Conserved Subunit Vaccine Candidates Conferring Cross‐Serotype Protection Against *Streptococcus suis* Serotypes 2, 7, 8, and 9

**DOI:** 10.1155/tbed/3394193

**Published:** 2026-04-28

**Authors:** Shun Kang, Hongkun Zhuang, Zeren Peng, Liye Chen, Jinlu Zhu, Zongfu Wu

**Affiliations:** ^1^ MOE Joint International Research Laboratory of Animal Health and Food Safety, College of Veterinary Medicine, Nanjing Agricultural University, Nanjing, 210014, China, njau.edu.cn; ^2^ Key Lab of Animal Bacteriology, Ministry of Agriculture and Rural Affairs, Nanjing, 210014, China, agri.gov.cn; ^3^ WOAH Reference Lab for Swine Streptococcosis, Nanjing, 210014, China

**Keywords:** reverse vaccinology, *Streptococcus suis*, subunit vaccine

## Abstract

*Streptococcus suis* is a major zoonotic pathogen with substantial serotype diversity and remains a serious threat to global swine production and public health. Accordingly, vaccines capable of inducing cross‐serotype protection are urgently needed. Here, we integrated comparative genomics, bioinformatics, and reverse vaccinology to identify five conserved candidate antigens from four clinically important *S. suis* serotypes (2, 7, 8, and 9), designated rP1–rP5. The previously reported antigen PstB was included as a reference control (rP6), as it has demonstrated protective efficacy against serotypes 2, 7, and 9. All six recombinant proteins were successfully expressed and purified, and in silico analyses supported their predicted antigenic features and immunogenic potential. Following three immunizations in mice, all candidates elicited robust antigen‐specific IgG responses. In opsonophagocytic assays, antisera against rP1, rP3, rP4, and rP6 displayed functional activity against serotypes 2, 7, 8, and 9. In a mouse infection model, vaccination with rP3, rP4, and rP6 reduced bacterial burdens in blood and multiple organs and conferred partial protection against all four serotypes, with rP4 producing a more pronounced reduction in tissue bacterial burdens than rP3 and rP6. Overall, rP4 and rP6 exhibited the most consistent cross‐serotype protection in both bacterial burden and survival analyses, whereas rP3 showed protective efficacy, particularly against serotype 2, and may serve as a useful component of a multivalent formulation. Collectively, these findings support the rational design of broadly protective, multicomponent subunit vaccines against *S. suis*.

## 1. Introduction

*Streptococcus suis* is a major pathogen that poses a serious threat to the swine industry, capable of causing meningitis, arthritis, septicemia, and other diseases in pigs [1]. It is also an important zoonotic pathogen, representing a significant risk to public health security. *S. suis* exhibits a high serotype diversity. Based on differences in capsular polysaccharide antigens, 29 serotypes (1–19, 21, 23–25, 27−31, and 1/2) have been identified [2], along with 34 novel classifications according to the *cps* locus (NCL1–20, 21a, 21b, 22–32, and Chz) [3–8]. Among these, 10 serotypes (1, 2, 4, 5, 7, 9, 14, 16, 24, and 31) have been reported to cause human infections [2, 7, 9].

*S. suis* serotype 2 (SS2) represents the most widespread and highly pathogenic lineage globally, accounting for the majority of human and swine infections [10]. It has been linked to several severe human epidemics, including outbreaks in Jiangsu (1998) and Sichuan (2005) in China, both of which led to substantial mortality [11, 12]. In addition to SS2, serotypes 7, 8, and 9 also carry clinical and epidemiological significance. Serotype 7 (SS7) circulates widely in pig herds throughout Europe, North America, and Thailand and has been confirmed to infect humans [13, 14]. Serotype 8 (SS8) ranks among the predominant strains isolated from diseased pigs in Spain, South Korea, Canada, and Brazil [10, 15–18]; 75% of representative strains display high virulence, pointing to a strong potential for causing severe clinical disease [19]. Serotype 9 (SS9) follows SS2 as the second most common serotype in global swine populations and is broadly distributed across Europe and Asia [20–23]. Its zoonotic nature is supported by reported cases of fatal septic shock in humans in Thailand [24]. Therefore, the development of effective preventive strategies against *S. suis* serotypes 2, 7, 8, and 9 represents an urgent research priority.

The overuse of antimicrobials has accelerated the emergence of antibiotic‐resistant *S. suis*, complicating clinical management and disease control in the field [25, 26]. Vaccination is, therefore, an increasingly important preventive option. Traditional inactivated vaccines can induce strong serotype‐specific antibody responses, but protection is usually limited to homologous strains, and cross‐protection against heterologous serotypes is often poor [27, 28]. This limitation has shifted attention toward conserved surface‐associated or secreted proteins as targets for cross‐protective subunit vaccines [29, 30]. Antigens with cross‐protective potential have been reported: PstB, a phosphate ABC transporter ATP‐binding protein, protected mice against SS2 (87.5%), SS7 (62.5%), and SS9 (87.5%) [31]. S‐ABC, a sugar ABC transporter substrate‐binding protein, provided protection against SS2 (87.5%), SS7 (50%), and SS9 (100%) [32]. MEAS1/MEAS3, epitope‐based vaccine constructs designed by reverse vaccinology, reduced histopathological damage and bacterial burdens and provided protection against SS2 (70%) and SS9 (80%) in mice [33]. Nevertheless, most antigens have been assessed against only a few serotypes, and their protection breadth across the diverse *S. suis* population remains limited [34–37]. Additional conserved protective antigens and vaccine strategies with improved multiserotype performance are still needed.

As an emerging strategy, reverse vaccinology employs genomics‐based approaches to enable high‐throughput screening of potential antigenic targets, followed by a systematic evaluation of their protective immunogenicity in animal models [38–40]. In this study, we integrated pan‐genome analysis with a reverse vaccinology strategy to prioritize conserved *S. suis* proteins shared across genetically diverse strains. Using this approach, five recombinant proteins (rP1–rP5) were selected as candidate vaccine components, while the previously reported antigen PstB was included as a reference control (rP6). These candidates were evaluated in a stepwise manner through in silico characterization, immunological assays, and cross‐serotype challenge experiments. Collectively, this work provides a systematic framework and prioritized antigen candidates to support the development of multicomponent subunit vaccines with improved cross‐serotype protection against *S. suis*.

## 2. Materials and Methods

### 2.1. Bacterial Strains and Culture

The virulent strains of *S. suis* serotypes 2, 7, 8, and 9 used in this study are shown in Table 1. All *S. suis* strains were streaked onto Todd–Hewitt Broth (THB; Hopebio, China) solid agar plates containing 5% sterile sheep blood and incubated at 37°C under 5% CO_2_. A single colony was selected and inoculated into the THB liquid medium, followed by incubation at 37°C with shaking at 180 rpm. *Escherichia coli* DH5α and BL21(DE3) strains were cultured on Luria–Bertani (LB) agar plates supplemented with 50 μg/mL kanamycin.

**Table 1:** The information of 16 *S. suis* virulent strains in this study.

Strain	Accession	Serotype	Host	Model evaluation
SC070731^a^	SAMN02603632	2	Diseased pig	Zebrafish and mice [9]
SC84	SAMEA2272312	2	Human	Mice [41]
05ZYH33	SAMN02603018	2	Human	Mice [42]
HA9801	SAMN00182532	2	Diseased pig	Mice [43]
ZY05719	SAMN02691834	2	Diseased pig	Mice [44]
WUSS013^a^	SAMN17982944	7	Diseased pig	Mice [45]
WUSS029	SAMN17982945	7	Healthy pig	Mice [45]
WUSS413	SAMN17982953	7	Healthy pig	Mice [45]
2018WUSS020	SAMN17982957	7	Healthy pig	Mice [45]
2019WUSS020	SAMN17982964	7	Healthy pig	Mice [45]
2018WUSS041	SAMN25761369	8	Healthy pig	Zebrafish and mice [19]
2018WUSS148^a^	SAMN25761373	8	Diseased pig	Zebrafish and mice [19]
2018WUSS151	SAMN29862018	8	Diseased pig	Zebrafish and mice [19]
GZ0565^a^	SAMN05567566	9	Diseased pig	Zebrafish [46]
WUSS010	SAMN40230529	9	Diseased pig	Zebrafish^b^
2018WUSS143	SAMN40230532	9	Diseased pig	Zebrafish and mice^b^

^a^Denotes strains included in the challenge experiment, whereas the remaining strains were analyzed only at the genomic level.

^b^Indicates unpublished data from our group.

### 2.2. Pan‐Genome Analysis

Functional annotation of the whole‐genome sequences for all strains was performed using Prokka (version 1.14) [47]. Subsequently, pan‐genome analysis was carried out with Roary (version 3.13.0), in which genes present in ≥95% of the strains were defined as the core genes [48]. The core genome sequences were then translated into the core proteome sequences using Prokka for further analysis.

### 2.3. Bioinformatic Characterization of Candidate Antigens

Systematic antigenicity prediction of the core proteome was conducted through the Vaxign2 (version 2.0) online platform [49], leading to the identification of proteins with high antigenicity scores. Subcellular localization prediction of the core proteome was conducted using PSORTb (version 3.0.3) and Gpos‐mPLoc [50, 51]. Proteins localized to the cell surface or secreted extracellularly were selected for further analysis. Subsequently, transmembrane domain prediction was carried out with TMHMM (version 2.0), and proteins containing no more than one transmembrane helix were retained [52]. This step aimed to enrich candidate antigen molecules with favorable solubility for subsequent investigations.

Adhesin prediction for the core proteome was performed using SPAAN [53], and candidate antigens with a high adhesin probability were selected. These proteins play a critical role in mediating initial bacterial adhesion and host–pathogen interactions. Subsequently, BLAST analysis was employed to compare the candidate antigens against the proteomes of humans, mice, and pigs. Ultimately, antigen molecules showing no significant homology with host proteins were selected to avoid potential autoimmune cross‐reactions and further evaluated in subsequent studies.

### 2.4. Docking Analysis of Candidate Antigens With Immune Receptors

To evaluate the binding potential between the candidate antigens and immune receptors, the three‐dimensional structure of the candidates were initially predicted using I‐TASSER (version 5.1) through homology modeling and fold recognition strategies, yielding preliminary structural models [54]. Subsequently, the models were refined and optimized via the Galaxy Refine to improve stereochemical quality and minimize steric clashes, thereby providing a reliable structural foundation for subsequent molecular docking analyses [55]. Finally, the refined models were evaluated using Ramachandran plots to assess the conformational rationality of their amino acid residues [56].

Molecular docking was conducted using the ClusPro 2.0 server between the candidate protein and human Toll‐like receptor 4 (human TLR4; PDB ID: 4g8a) as well as human major histocompatibility complex class II (human MHC II; PDB ID: 5jlz) [57]. The resulting docking poses were visualized using PyMOL (version 2.5) to depict molecular interaction patterns and binding interface architectures. Interfacial interactions, including hydrogen bonds, hydrophobic contacts, and specific residue involvements, were further characterized via PDBsum [58].

### 2.5. In Silico Immunological Profiling

To evaluate the potential immune response induced by the candidate protein, we performed immune simulation using the C‐IMMSIM server [59]. Three antigen injections were simulated at time steps 6, 48, and 90, corresponding to Days 2, 16, and 30, respectively, with each time step representing 8 h of real time. The simulation volume was set to 10, and the total number of simulation steps was 540. Representative HLA alleles, including HLA‐A0201, HLA‐B0702, and HLA‐DRB1_0101, were used for the simulation. All other parameters were kept at their default settings.

### 2.6. Expression and Purification of Recombinant Proteins

Based on the candidate protein sequences of SS2 strain SC070731, gene‐specific primers were designed to amplify the target fragment by polymerase chain reaction (PCR), which was subsequently cloned into the pET‐28a vector using homologous recombination; the correctness of the constructed plasmids were confirmed by sequencing before being transformed into *E. coli* BL21(DE3)‐competent cells for recombinant protein expression. The transformed bacteria were grown in LB medium containing 50 μg/mL kanamycin to mid‐log phase, induced with 1 mM isopropyl beta‐D‐1‐thiogalactopyranoside (IPTG), and incubated at 16°C with shaking at 120 rpm for 12–16 h; after harvesting, the cells were lysed by ultrasonication, and the recombinant proteins rP1–rP6 were purified by HisTrap HP column (GE Healthcare, USA). Protein expression was validated by 12% SDS‐PAGE (Vazyme, Nanjing, China), and the concentration of the purified protein was quantified with the bicinchoninic acid (BCA) assay (Thermo Scientific, USA). Primers are provided in Supporting Information 1: Table S1.

### 2.7. Western Blot Analysis

The specificity of the recombinant proteins was verified by Western blot. The purified candidate antigens (rP1–rP6) were separated by 12% SDS‐PAGE and subsequently transferred to a polyvinylidene fluoride (PVDF) membrane (Bio‐Rad, USA) using a semidry transfer system. The membrane was blocked at 25°C for 2 h with 5% skim milk in Tris‐buffered saline. After blocking, the membrane was incubated overnight at 4°C with a mouse anti‐His tag monoclonal antibody (Abmart, China) diluted at 1:5000. Following primary antibody incubation, the membrane was washed three times for 10 min each with PBST buffer containing 0.05% Tween‐20. Then, it was incubated for 1 h at room temperature with a horseradish peroxidase (HRP)‐conjugated goat anti‐mouse IgG (Beyotime, China) secondary antibody diluted at 1:5000, followed by three additional washes with PBST for 10 min each. Finally, the immunoreactive bands were detected using an enhanced chemiluminescence (ECL) reagent kit (Vazyme, China).

### 2.8. Mouse Immunization and Antibody Titer Determination

Five‐week‐old specific‐pathogen‐free (SPF) female Institute of Cancer Research (ICR) mice were used to assess antibody responses to the candidate antigens. Mice were randomly assigned to seven groups (six immunization groups and one PBS–adjuvant control), with three mice per group (*n* = 3). To confirm the absence of preexisting antibodies against *S. suis*, three mice were randomly selected for blood sampling on Day 0. Antigens were prepared by emulsifying each recombinant protein with the ISA 206V adjuvant (SEPPIC, France) at a 1:1 (v/v) ratio by vortexing. Mice were immunized subcutaneously at multiple sites (0.2 mL per mouse) on Days 2, 16, and 30. The priming dose contained 100 μg of recombinant protein, and each booster contained 50 μg. Blood samples were collected on Days 15, 29, and 43. At each time point, ~100 μL of blood was obtained from the orbital venous plexus, kept at 4°C overnight, and centrifuged to separate sera, which were stored until the analysis.

Specific IgG titers were determined by indirect ELISA. Briefly, 96‐well plates were coated overnight with recombinant proteins (rP1–rP6; 10 μg/mL). After blocking with 2% bovine serum albumin (BSA), sera from each group were diluted in 0.5% BSA starting at 1:200 and then serially diluted twofold. Bound IgG was detected with HRP‐conjugated goat anti‐mouse IgG (Beyotime, China) and developed with TMB (Sigma, USA). The absorbance was measured at 450 nm. Endpoint titers were defined as the highest dilution meeting the positivity criterion, with *P*/*N* ≥ 2.1 (OD _positive_/OD _negative_) used as the cutoff.

### 2.9. Opsonophagocytic Assay

For the opsonophagocytic assay, mice were immunized according to the procedure described in Section 2.8, with five mice in each group (*n* = 5). Blood was collected 14 days after the third immunization to prepare immune sera. *S. suis* was cultured in THB (Hopebio, China) to mid‐log phase, washed twice with sterile PBS, and adjusted to 1 × 10^7^ colony‐forming unit (CFU)/mL. Immune serum (100 μL) or negative control serum (100 μL) was mixed 1:1 with the bacterial suspension and preincubated at 37°C for 30 min. Fresh swine whole blood (800 μL) was then added, and the mixtures were incubated at 37°C.

Aliquots were collected at 1, 2, and 3 h, serially diluted in sterile PBS, plated on THB agar, and incubated for colony enumeration. At each time point, the survival rate in each immune serum group was calculated relative to that in the adjuvant control group, with the adjuvant control group defined as 100%, to assess antibody‐mediated opsonophagocytic activity.

### 2.10. Bacterial Burden and Survival Analysis

The protective efficacy of candidate antigens was evaluated using 5‐week‐old SPF female ICR mice. For the organ bacterial burden assay, mice were randomly divided into five groups (four protein‐immunized groups and one PBS–adjuvant control), with five mice per group (*n* = 5; total = 25). Fourteen days after the third immunization, mice were intraperitoneally challenged with SS2 strain SC070731 (8 × 10^8^ CFU/mouse). At 12 h postchallenge, mice were euthanized, and blood, brain, spleen, and liver samples were aseptically collected. Samples were serially diluted and plated on THB agar, and bacterial loads were determined by CFU enumeration.

To assess cross‐serotype protective efficacy, mice were assigned to four vaccination groups (three immunized groups and one PBS–adjuvant control). Each vaccination group was further subdivided into four challenge subgroups and inoculated with *S. suis* serotypes 2, 7, 8, or 9, with 10 mice per subgroup (*n* = 10; total = 160). Intraperitoneal challenges were performed with SS2 strain SC070731 (1.6 × 10^9^ CFU/mouse), SS7 strain WUSS013 (6 × 10^8^ CFU/mouse), SS8 strain 2018WUSS148 (5 × 10^8^ CFU/mouse), or SS9 strain GZ0565 (6 × 10^8^ CFU/mouse). These challenge strains, including SC070731 [9], WUSS013 [45], 2018WUSS148 [19], and GZ0565 [46], were previously characterized as highly virulent, and each caused mortality rates exceeding 80% in both zebrafish and mouse infection models. Mice were monitored daily for 7 days, and survival was recorded to evaluate vaccine‐induced protection.

### 2.11. Statistical Analysis

For experiments involving three or more groups, comparisons were made using one‐way ANOVA, followed by Dunnett’s multiple‐comparisons test. Comparisons between two groups were analyzed using the unpaired *t*‐test. All statistical analyses were performed using GraphPad Prism version 8.0. Data were expressed as mean ± standard deviation (SD), and a *p*‐value < 0.05 was considered statistically significant.

### 2.12. Ethical Statement

Five‐week‐old female SPF ICR mice were purchased from Shanghai SLAC Laboratory Animal Co., Ltd. All animal experiments were conducted at the Laboratory Animal Center of Nanjing Agricultural University in accordance with the animal welfare guidelines of the Animal Research Committee of Jiangsu Province and were approved by the Institutional Ethics Committee (Approval IDs: NJAU.No20240308039 and NJAU.No20241008196). Blood samples were collected under ketamine and xylazine anesthesia. Mice were euthanized by cervical dislocation after anesthesia, and every measure was taken to reduce distress and minimize suffering throughout the study. Anesthetic and euthanasia procedures were conducted in compliance with the American Veterinary Medical Association (AVMA) standards for animal euthanasia (2020).

## 3. Results

### 3.1. Identification of Conserved Candidate Antigens

**Figure 1:**
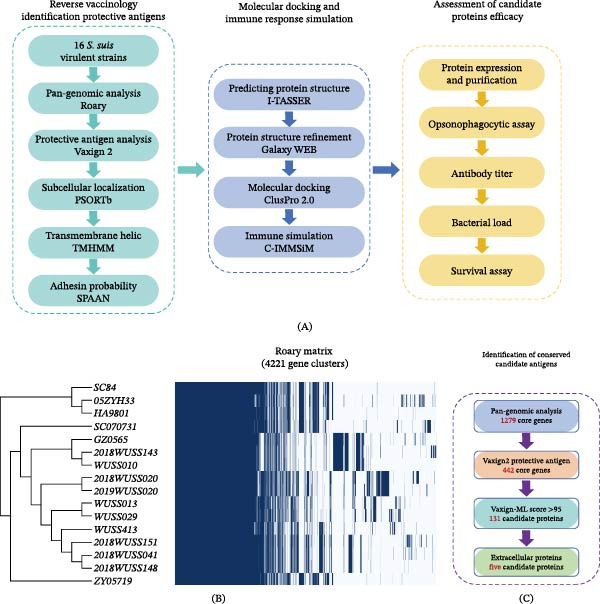
Identification of conserved candidate antigens in *S. suis*. (A) Schematic overview of the reverse vaccinology‐based strategy used for systematic antigen discovery. A pangenome‐guided bioinformatic screening pipeline was applied to identify conserved candidate antigens, followed by experimental validation of their protective efficacy using in vitro assays and in vivo challenge models. (B) Core‐genome phylogenetic tree and gene presence/absence matrix of 16 *S. suis* virulent strains. The left panel depicts strain relatedness inferred from core‐genome analysis, while the right panel shows the distribution of gene clusters across genomes. (C) Stepwise filtering of the core proteome to identify conserved vaccine candidates. Starting from 1279 core genes, sequential screening based on predicted protective potential and subcellular localization progressively narrowed the candidates to a final set of extracellular proteins selected for further evaluation.

To identify conserved antigens with cross‐protective potential against *S. suis* serotypes 2, 7, 8, and 9, we applied a reverse vaccinology strategy to analyze 16 virulent strains (Figure 1A). Pan‐genome analysis revealed 1279 core genes shared across all strains, which were translated into protein sequences for subsequent screening (Figure 1B).

From the core proteome, candidate antigens were initially prioritized based on their predicted protective potential. Proteins with extracellular localization or high adhesin probability were subsequently selected for further evaluation. Among the top‐ranking candidates, five newly identified proteins were successfully expressed and purified and were designated rP1–rP5. To provide a reference for comparison, rP6, corresponding to PstB, a phosphate ABC transporter ATP‐binding protein involved in inorganic phosphate uptake, was included as a positive control. Recombinant PstB has previously been shown to confer 87.5% protection against lethal challenge with *S. suis* serotypes 2 and 9 and 62.5% protection against serotype 7 [31]. Together, these six recombinant proteins (rP1–rP6) were advanced for subsequent experimental evaluation (Figure 1C and Table 2).

**Table 2:** Characteristics of candidate antigens evaluated in this study.

Protein ID	Annotation	Vaxign2 score	Accession	Source
rP1	Extracellular solute‐binding protein	98.51	WP_002936107.1	In the study
rP2	Peptidoglycan hydrolase PcsB	95.51	WP_012774880.1	In the study
rP3	Putative efflux‐associated membrane protein	99.63	WP_012775008.1	In the study
rP4	DUF1002 domain‐containing protein	97.11	WP_011921733.1	In the study
rP5	ABC transporter substrate‐binding protein	97.82	WP_012774915.1	In the study
rP6	Phosphate ABC transporter ATP‐binding protein PstB	81.45	WP_002940866.1	[31], as a control

### 3.2. Molecular Docking Analysis of Candidate Antigens With Immune Receptors

To explore the potential interactions between the candidate antigens and host immune receptors at the bioinformatics level, we performed homology modeling and docking analyses for all six proteins (rP1–rP6) against human TLR4 and human MHC class II (Supporting Information 2: Figure S1). Detailed modeling parameters for the candidate proteins are listed in Supporting Information 1: Table S2, and the docking parameters are listed in Supporting Information 1: Table S3. TLR4 is a well‐characterized pattern‐recognition receptor that detects conserved microbial signals and triggers early innate immune responses [60, 61], whereas MHC class II mediates antigen presentation to CD4^+^ T cells and supports the development of adaptive immunity [62, 63].

All six proteins produced computationally plausible docking poses with both human TLR4 and human MHC class II, and the top‐ranked models suggested potential binding interfaces. Interface analysis indicated that these predicted complexes involved multiple types of interactions, including hydrogen bonds, salt bridges, and nonbonded contacts (Supporting Information 3: Figure S2 and Supporting Information 4: Figure S3). These in silico results suggest that the proteins may engage these receptors through surface‐exposed regions. They were used to provide a preliminary structural basis for subsequent immunogenicity evaluation.

### 3.3. In Silico Immune Simulation of Candidate Antigens

To obtain an initial indication of immunogenicity, we carried out in silico immune simulations for the six candidate antigens (rP1–rP6) using a three‐dose vaccination schedule over a 180‐day period. The simulations predicted that each antigen could induce a measurable humoral response, with antigen‐specific IgG increasing after the primary dose. Predicted titers further rose after each booster and remained at relatively high levels for the remainder of the simulation (Supporting Information 5: Figure S4).

In parallel, the model suggested an expansion of B‐cell populations and a sustained memory B‐cell response, which is consistent with the development of longer‐term immune recall (Supporting Information 6: Figure S5). Overall, these results support the view that rP1–rP6 have the potential to trigger robust antibody responses and maintain immune memory, providing a rationale for subsequent experimental validation.

### 3.4. Expression and Purification of Candidate Antigens

**Figure 2:**
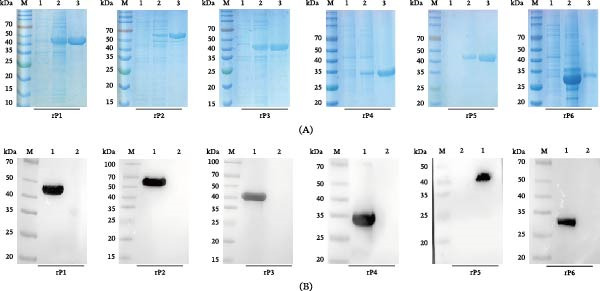
Recombinant expression and purification of candidate antigens. (A) Representative 12% SDS‐PAGE showing soluble expression of the recombinant proteins following low‐temperature induction. Lane 1, uninduced cell lysate; Lane 2, induced lysate; Lane 3, purified recombinant antigen. (B) Western blot confirming the immunoreactivity of the purified proteins using an anti‐His tag antibody. Lane 1, purified recombinant protein; Lane 2, empty pET‐28a vector control. M indicates the protein molecular weight marker.

Recombinant proteins corresponding to the candidate antigens (rP1–rP6) were successfully obtained for downstream immunization studies. SDS‐PAGE analysis showed markedly enhanced expression of the recombinant proteins after induction compared to the uninduced control (Figure 2A). Western blot analysis further confirmed the presence of distinct single bands at the expected molecular weights of 45.6 kDa, 45.1 kDa, 39.7 kDa, 34.3 kDa, 48.6 kDa, and 34.2 kDa, respectively (Figure 2B). Overall, the recombinant proteins were purified to high purity and were therefore suitable for subsequent animal experiments.

### 3.5. Immunogenicity Evaluation of Candidate Antigens in Mice

**Figure 3:**
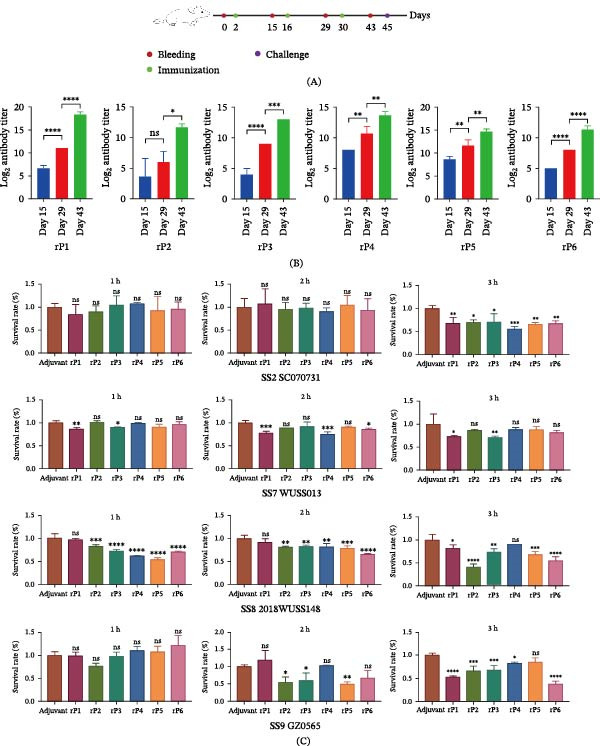
In vitro immunological evaluation of candidate antigens. (A) Immunization and sampling scheme, including three antigen–adjuvant immunizations (green circles; Days 2, 16, and 30), serum collections (red circles; Days 0, 15, 29, and 43), and the final challenge (blue circle; Day 45). (B) Antigen‐specific serum IgG titers measured by ELISA on Days 15, 29, and 43. Data were analyzed using one‐way ANOVA followed by Dunnett’s multiple‐comparisons test. (C) Opsonophagocytic activity of immune sera against SS2 strain SC070731, SS7 strain WUSS013, SS8 strain 2018WUSS148, and SS9 strain GZ0565 assessed using fresh swine whole blood. Viable bacteria were quantified at 1, 2, and 3 h postincubation, and survival was expressed relative to the adjuvant control serum. Data were analyzed using one‐way ANOVA followed by Dunnett’s multiple‐comparisons test, with each recombinant‐protein group compared with the adjuvant control group at the corresponding time point for each strain. Data are presented as mean ± SD. Asterisks indicate statistically significant differences versus the adjuvant control group “ ^∗^”, “ ^∗∗^”, “ ^∗∗∗^”, and “ ^∗∗∗∗^” indicate *p* < 0.05, *p* < 0.01, *p* < 0.001, and *p* < 0.0001, respectively; ns, not significant).

The immunogenicity of the six recombinant proteins (rP1–rP6) was assessed in mice following the immunization scheme shown in Figure 3A. Antigen‐specific IgG was quantified by an ELISA. Across all groups, IgG responses increased progressively with each dose. After the third immunization, endpoint titers for all proteins exceeded 1:2^10^ and were higher than those measured after the second immunization (Figure 3B). Together, these data show that rP1–rP6 induce clear antigen‐specific humoral responses in mice.

### 3.6. Functional Opsonophagocytic Activity of Immune Sera Across Serotypes

The opsonophagocytic activity of immune sera raised against rP1–rP6 was evaluated using a swine whole‐blood assay, with bacterial survival monitored over time (Figure 3C). Sera against rP1, rP3, rP4, and rP6 exhibited significant opsonophagocytic killing against all four *S. suis* serotypes tested, indicating the induction of broadly functional antibody‐mediated clearance. Although the magnitude and kinetics of killing varied among serotypes, opsonophagocytic activity was detectable at at least one time point within the 1–3 h incubation period. For serotypes 2 and 9, opsonophagocytic killing was predominantly observed at later time points, whereas for serotype 8, functional activity emerged earlier and was more sustained, particularly for rP3 and rP6. In contrast, sera raised against rP2 and rP5 did not show significant opsonophagocytic activity against serotype 7 and were therefore not advanced to subsequent *in vivo* challenge experiments.

### 3.7. Bacterial Burden and Survival Analysis

**Figure 4:**
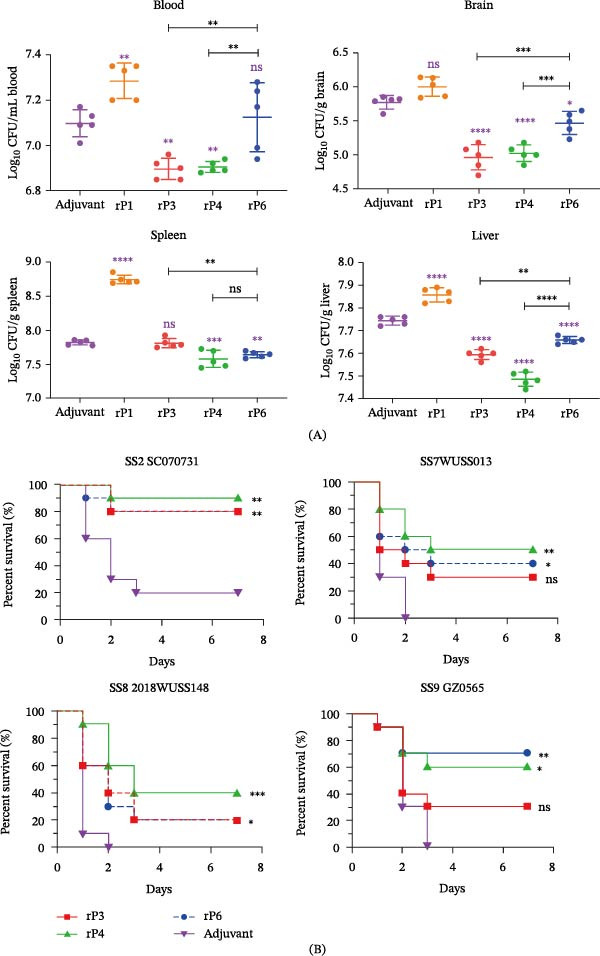
Bacterial Burden and Survival Analysis. (A) Bacterial loads in blood, spleen, brain, and liver collected 12 h after challenge with *S. suis* strain SC070731. Burdens are expressed as CFU per milliliter of blood or CFU per gram of tissue. Purple asterisks denote comparisons versus the adjuvant control group. Black asterisks (with brackets) denote statistical comparisons between the indicated pair of groups. Data were analyzed using one‐way ANOVA followed by Dunnett’s multiple‐comparisons test. (B) Survival of immunized mice (*n* = 10 per group) following challenge with virulent *S. suis* strains SC070731, WUSS013, 2018WUSS148, or GZ0565. Animals were monitored for 7 days postchallenge. Survival curves were analyzed using the log‐rank (Mantel‐Cox) test. “ ^∗^”, “ ^∗∗^”, “ ^∗∗∗^”, and “ ^∗∗∗∗^” indicate *p* < 0.05, *p* < 0.01, *p* < 0.001, and *p* < 0.0001, respectively; ns, not significant.

To determine whether immunization with rP1, rP3, rP4, or rP6 enhances bacterial clearance during *S. suis* infection, mice were immunized and subsequently challenged intraperitoneally with the virulent serotype 2 strain SC070731. Bacterial burdens in the blood, brain, spleen, and liver were quantified 12 h postchallenge (Figure 4A). Compared with the adjuvant control, rP3 and rP4 significantly reduced bacterial burdens in the blood, whereas rP6 showed no significant effect; both rP3 and rP4 also resulted in significantly lower blood bacterial loads than rP6. In the brain, all three candidates reduced bacterial counts relative to the control, with rP3‐ and rP4‐immunized mice exhibiting significantly lower burdens than those receiving rP6. In the spleen, bacterial reduction was antigen‐dependent, with rP4 and rP6 showing significant effects, while rP3 did not. In the liver, rP3, rP4, and rP6 all reduced bacterial loads compared with the control, with rP3 and particularly rP4 achieving greater clearance than rP6. In contrast, rP1 did not promote bacterial clearance and was associated with higher bacterial burdens in multiple organs. Collectively, these data indicate that rP3, rP4, and rP6 enhance the early control of SS2 infection, with rP4 showing the most consistent reduction across tissues.

The protective efficacy was further evaluated in survival analysis using *S. suis* serotypes 2, 7, 8, and 9 (Figure 4B). In adjuvant‐treated mice, SS2 challenge resulted in ~20% survival, whereas challenges with serotypes 7, 8, and 9 were uniformly lethal. Immunization with recombinant proteins improved survival in a serotype‐dependent manner. Following the SS2 challenge, rP3, rP4, and rP6 all conferred significant protection, with final survival rates of ~80% for rP3 and rP6 and 90% for rP4. After the SS7 challenge, rP4 and rP6 significantly increased survival to 50% and 40%, respectively, whereas rP3 provided limited protection (30%) and did not differ significantly from the control. Overall protection against SS8 was modest but statistically significant compared with the adjuvant control group, with rP4 achieving the highest survival (40%), while rP3 and rP6 conferred a lower but measurable benefit (20%). In the SS9 model, rP4 and rP6 again provided significant protection, ending at 60% and 70% survival, respectively, whereas rP3 remained comparable to the control. Across the serotypes 2, 7, and 8 tested, no statistically significant differences were detected among the rP3‐, rP4‐, and rP6‐immunized groups; for serotype 9, no statistically significant differences were detected between rP4‐ and rP6‐immunized groups.

Taken together, rP4 and rP6 displayed the most consistent cross‐serotype protection in both bacterial burden and survival analyses, whereas rP3 showed strong protection against SS2 (80% survival) but limited efficacy against heterologous serotypes.

## 4. Discussion

A major challenge in the development of effective *S. suis* vaccines is the extensive serotype diversity and the limited cross‐protective immunity observed among the circulating serotypes. Conserved surface‐associated and secreted proteins are therefore considered promising targets for broad‐spectrum subunit vaccines [29, 30]. However, most available protection data are derived from serotype 2 challenge models, and systematic evaluation against other prevalent serotypes remains limited. In this context, our study extends protective assessment to serotypes 2, 7, 8, and 9 and provides evidence that conserved proteins can confer cross‐serotype protection. Using an integrated pan‐genomic and reverse vaccinology approach, we identified and evaluated a focused set of conserved protein candidates with potential application in the development of multivalent *S. suis* subunit vaccines.

A homologous pneumococcal protein corresponding to rP1 has been reported to induce protective immunity following intranasal administration in a colonization model [64]. A five‐protein subunit formulation that included rP1 significantly reduced severe disease in a pig model and achieved 83.3% protection against a serotype 2 strain challenge [65]. However, in this study, we found that the higher bacterial loads observed in the blood, spleen, and liver of rP1‐immunized mice, relative to those of the adjuvant control group, were unexpected. At present, the mechanism underlying this finding remains unclear. One possibility is that rP1 immunization alone induced an immune response that was not only nonprotective but also functionally unfavorable for bacterial clearance in vivo. For example, rP1 alone may have elicited antibodies or other immune responses that interfered with effective opsonophagocytic clearance or altered host immune responses in a manner that was detrimental to bacterial control.

rP2 is a CHAP‐domain membrane protein annotated as an amidase and has previously been identified as an immunoreactive protein across prevalent *S. suis* serotypes by comparative immunoproteomic analyses [66]. Its pneumococcal homolog, PcsB (99% query coverage; 57.11% identity), is a conserved cell‐division protein that can elicit opsonic antibodies and confer cross‐serotype protection in murine models, supporting the relevance of this functional class as potential vaccine targets [67–69]. rP5 is a predicted surface‐associated sugar ABC transporter substrate‐binding protein that was prioritized primarily based on its high in silico score and predicted membrane localization. Although sera raised against rP2 and rP5 exhibited opsonophagocytic activity against serotypes 2, 8, and 9, no significant activity was observed against serotype 7. Given that opsonophagocytic activity is a key correlate for evaluating vaccine efficacy against *S. suis*, rP2 and rP5 were not advanced to subsequent in vivo challenge experiments.

rP3 is predicted to be involved in membrane‐associated processes or transport and has been detected in both membrane vesicle and surface proteomes of *S. suis* clinical isolates, consistent with potential antibody accessibility [70, 71]. In line with this localization, rP3 conferred strong protection against serotype 2 but showed limited efficacy against heterologous serotypes, suggesting that its protective capacity may be partially serotype‐dependent.

rP4 is a DUF1002‐domain protein that has been experimentally confirmed to be surface‐localized in *S. suis*[71]. Notably, a DUF1002‐containing antigen from *Streptococcus iniae* (97% query coverage; 30.23% identity) has also been reported to confer in vivo protection in a fish challenge model, supporting the broader protective potential of this protein family [72]. rP6 corresponds to PstB, the ATP‐binding component of a phosphate ABC transporter, and was included as a reference antigen. A previous study demonstrated its protective efficacy against multiple *S. suis* serotypes in mice [31]. Consistent with these observations, rP4 and rP6 exhibited the most consistent cross‐serotype protection in both bacterial burden and survival analyses in the present study.

Following the challenge with serotype 2, immunization with rP3, rP4, and rP6 conferred clear protective effects. In contrast, overall protection against serotypes 7 and 8 was more limited, with only partial protection observed for a subset of candidates, whereas the protection conferred by rP3 against serotype 9 was low. One possible explanation for these differences is variation in the degree of surface exposure of protective antigens among strains of different serotypes, which may affect antibody accessibility and recognition. Another contributing factor may be the relatively high challenge doses used in the infection models, which likely accelerated disease progression and limited the window for effective immune control; for example, in the serotype 8 challenge model, ~90% of control mice succumbed within the 1st day postinfection. Therefore, future studies should prioritize the optimization of challenge doses and antigen combinations to enhance the overall protective efficacy. In particular, rP3, rP4, and rP6 could be combined with one another or paired with additional conserved antigens to recruit complementary immune mechanisms and broaden serotype coverage. These proteins could also be incorporated into an inactivated whole‐cell vaccine strategy to combine antigen‐specific immunity with broader immune stimulation, potentially further improving heterologous protection. In parallel, optimization of immunization routes, adjuvant selection, and antigen delivery systems will be essential to maximize vaccine‐induced protection.

It should also be noted that although the mouse immunization and challenge model provides a practical platform for the preliminary screening of vaccine candidates, mice are not the natural host of *S. suis*, and protective efficacy observed in mice may not fully predict vaccine performance in pigs. Future studies should further evaluate the most promising candidates, particularly rP4 and rP6, in piglet immunization and challenge models.

## 5. Conclusion

This study establishes a systematic framework for the identification of cross‐protective antigens in *S. suis* and evaluates the protective potential of six candidate antigens. Notably, rP4, newly identified in this study, emerges as a strong candidate for broadly protective subunit vaccine development. rP3 also shows protective efficacy, particularly against serotype 2, and may contribute to cross‐serotype protection as part of a multicomponent formulation. Collectively, these findings provide experimental evidence and identify prioritized antigen candidates to support the rational design of broadly protective vaccines against *S. suis*.

## Author Contributions

Data curation: Shun Kang, Zeren Peng, and Zongfu Wu. Formal analysis: Shun Kang, Hongkun Zhuang, Jinlu Zhu, and Zongfu Wu. Investigation: Shun Kang, Liye Chen, and Zongfu Wu. Project administration, supervision: Zongfu Wu. Writing – original draft: Shun Kang, Jinlu Zhu, and Zongfu Wu .Writing – review and editing: Shun Kang and Zongfu Wu

## Funding

This work was supported by the National Natural Science Foundation of China (Grant 32573366).

## Conflicts of Interest

The authors declare no conflicts of interest.

## Supporting Information

Additional supporting information can be found online in the Supporting Information section.

## Supporting information


**Supporting Information 1** Table S1: Primers information. Table S2: Key structural modeling parameters of candidate antigens. Table S3: Docking results of candidate antigens with immune receptors.

**Supporting Information 2** Figure S1: Predicted three‐dimensional structures of candidate antigens and host receptors. Ribbon representations of the modeled proteins are shown as follows: (A) rP1, (B) rP2, (C) rP3, (D) rP4, (E) rP5, (F) rP6, (G) human TLR4, and (H) human MHC class II.

**Supporting Information 3** Figure S2: Molecular docking of candidate antigens with the human immune receptor TLR4. Representative docking models of human TLR4 in complex with each recombinant antigen are shown: (A) rP1, (B) rP2, (C) rP3, (D) rP4, (E) rP5, and (F) rP6. Dashed boxes indicate the predicted interaction interfaces, which are enlarged in the lower panels; residues involved in the interface are listed on the right.

**Supporting Information 4** Figure S3: Molecular docking of candidate antigens with the human immune receptor MHC class II. Representative docking models of human MHC II in complex with each recombinant antigen are shown: (A) rP1, (B) rP2, (C) rP3, (D) rP4, (E) rP5, and (F) rP6. Dashed boxes indicate the predicted binding interfaces, which are enlarged in the lower panels; residues involved in the interface are listed on the right.

**Supporting Information 5** Figure S4: Simulated immune responses induced by candidate antigens. In silico immunization profiles show the predicted kinetics of antigen levels and antibody responses, including IgM, IgG, and IgG subclasses, following repeated antigen exposures for each candidate: (A) rP1, (B) rP2, (C) rP3, (D) rP4, (E) rP5, and (F) rP6.

**Supporting Information 6** Figure S5: Predicted B‐cell dynamics following simulated immunization with the candidate antigens. In silico immune simulations depict the kinetics of total B cells, nonmemory B cells, memory B cells, and isotype‐specific B‐cell responses over time for each candidate antigen: (A) rP1, (B) rP2, (C) rP3, (D) rP4, (E) rP5, and (F) rP6.

## Data Availability

All sequencing data analyzed in this study were obtained from the National Center for Biotechnology Information (NCBI) public repository. Accession numbers are provided in Table 1. Additional supporting information can be found in the Supporting Information of this article.
