# Supplementary material for: Identification and Evaluation of Conserved Subunit Vaccine Candidates Conferring Cross‐Serotype Protection Against Streptococcus suis Serotypes 2, 7, 8, and 9
Source: Transbound Emerg Dis. 2026 Apr 28;2026:3394193. doi: 10.1155/tbed/3394193 (PMC13125869; doi:10.1155/tbed/3394193)
Supplement: Supplementary file 1 — Supporting Information 1 Table S1: Primers information. Table S2: Key structural modeling parameters of candidate antigens. Table S3: Docking results of candidate antigens with immune receptors. [file TBED-2026-3394193-s006.docx]

Table S1 Primers information.

| Protein | Primer | Sequence (5'-3') |
| --- | --- | --- |
| rP1 | rP1-BamHI-F | cagcaaatgggtcgcggatccTCAAACTCAGGTTCATCAACAGAGG |
|  | rP1-HindIII-R | ctcgagtgcggccgcaagcttCTAGTTGCTGTGTTTTTGAGCAATT |
| rP2 | rP2-BamHI-F | cagcaaatgggtcgcggatccGATGTAGATAGTCAGATTGCAACAAAAA |
|  | rP2-HindIII-R | ctcgagtgcggccgcaagcttTTAATATGGAGGGTAAATGTAGTAAACCG |
| rP3 | rP3-BamHI-F | cagcaaatgggtcgcggatccGATGTAGCCATGAGTATGACCTATACTG |
|  | rP3-HindIII-R | ctcgagtgcggccgcaagcttCTAATAAGCTGGTTCTTCATTGGCT |
| rP4 | rP4-BamHI-F | cagcaaatgggtcgcggatccATTCAAACAGATGTTATCAATGAAAAATG |
|  | rP4-XhoI-R | gtggtggtggtggtgctcgagTTATGCAAATAGACCGGTCAAGC |
| rP5 | rP5-BamHI-F | cagcaaatgggtcgcggatccTCTACTTCGACTGAGTCTAGCAAAACT |
|  | rP5-HindIII-R | ctcgagtgcggccgcaagcttCTATTTTTCAGTTGCTAATTCAGCATT |
| rP6 | rP6-EcoRI-F | atgggtcgcggatccgaattcATGGCAGATTACAACTGGAATGAA |
|  | rP6-HindIII-R | ctcgagtgcggccgcaagcttCTATCCAAAATGTCCTGAAACGTAGT |

Table S2 Key structural modeling parameters of candidate antigens.

| Protein | C-score | Estimated TM-score | Estimated RMSD |
| --- | --- | --- | --- |
| rP1 | -0.69 | 0.63 ± 0.14 | 8.5 ± 4.5 |
| rP2 | -1.57 | 0.52 ± 0.15 | 10.6 ± 4.6 |
| rP3 | -1.59 | 0.52 ± 0.15 | 10.4 ± 4.6 |
| rP4 | -2.64 | 0.41 ± 0.14 | 12.6 ± 4.3 |
| rP5 | -0.76 | 0.62 ± 0.14 | 8.7 ± 4.5 |
| rP6 | 0.59 | 0.79 ± 0.09 | 4.7 ± 3.1 |

Note: TM-score and RMSD represent the estimated values provided by I-TASSER for the top-ranked model of each protein.

Table S3 Docking results of candidate antigens with immune receptors.

| Receptor | Candidate Antigen | Cluster Members | Cluster Cente Weighted Score |
| --- | --- | --- | --- |
| TLR4 | rP1 | 103 | -613.7 |
| TLR4 | rP2 | 59 | -763.9 |
| TLR4 | rP3 | 114 | -816.7 |
| TLR4 | rP4 | 191 | -682.6 |
| TLR4 | rP5 | 71 | -538.0 |
| TLR4 | rP6 | 113 | -914.3 |
| MHC II | rP1 | 93 | -615.2 |
| MHC II | rP2 | 68 | -1022.0 |
| MHC II | rP3 | 142 | -904.7 |
| MHC II | rP4 | 71 | -784.1 |
| MHC II | rP5 | 93 | -569.0 |
| MHC II | rP6 | 107 | -962.5 |
