# Supplementary figures and images for: Identification and Evaluation of Conserved Subunit Vaccine Candidates Conferring Cross‐Serotype Protection Against Streptococcus suis Serotypes 2, 7, 8, and 9
Source: Transbound Emerg Dis. 2026 Apr 28;2026:3394193. doi: 10.1155/tbed/3394193 (PMC13125869; doi:10.1155/tbed/3394193)

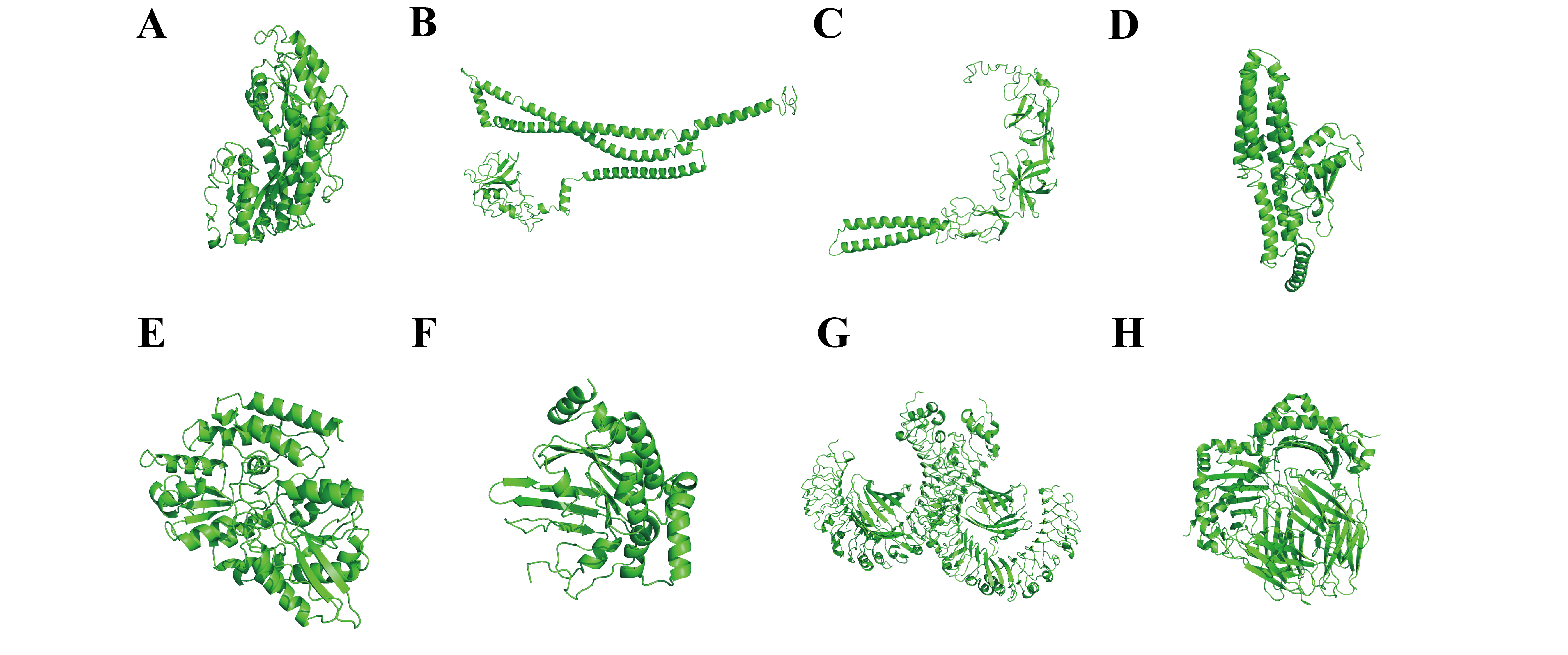

Supplement: Supplementary file 2 — Supporting Information 2 Figure S1: Predicted three‐dimensional structures of candidate antigens and host receptors. Ribbon representations of the modeled proteins are shown as follows: (A) rP1, (B) rP2, (C) rP3, (D) rP4, (E) rP5, (F) rP6, (G) human TLR4, and (H) human MHC class II. [file TBED-2026-3394193-s004.tif]

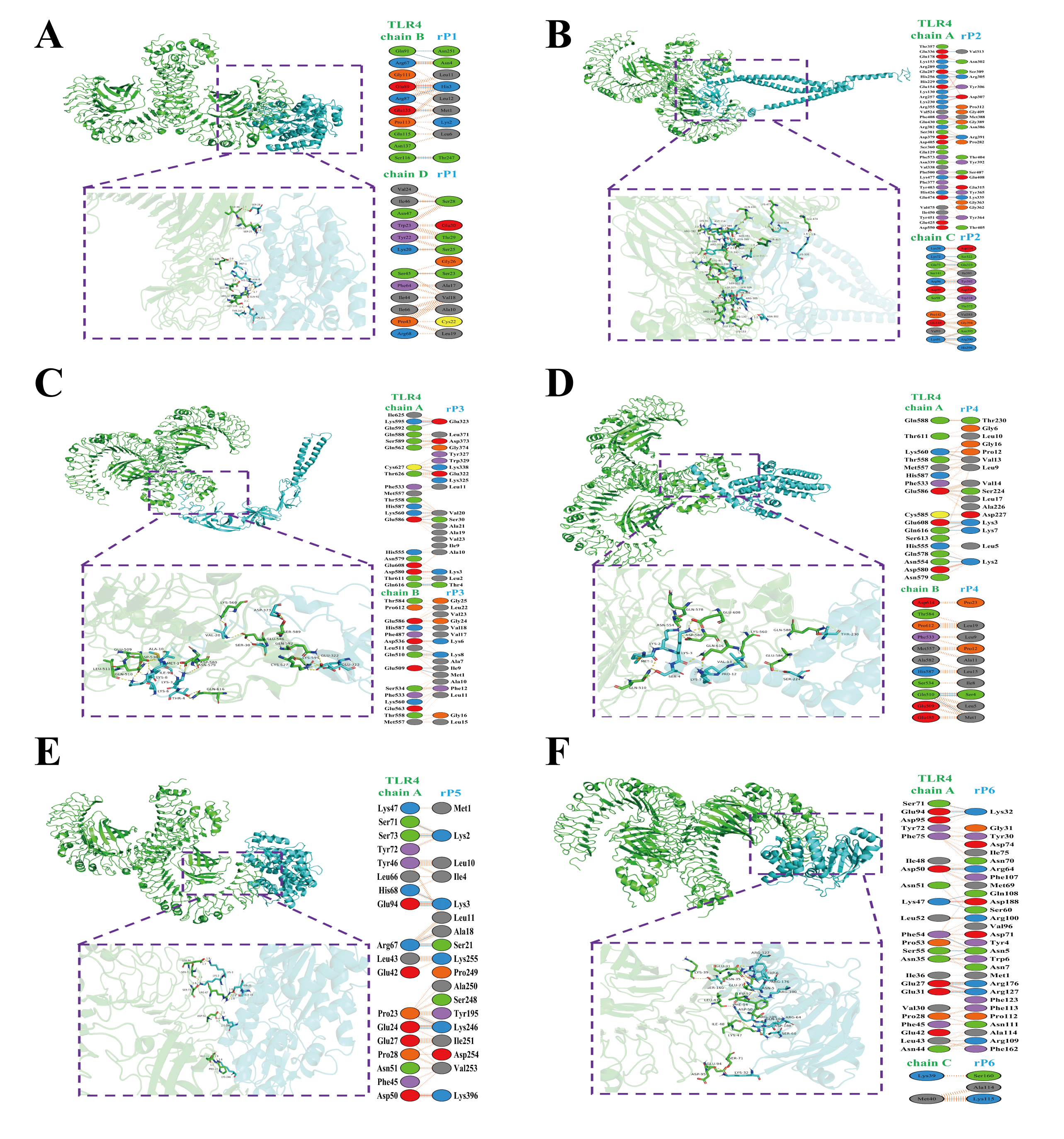

Supplement: Supplementary file 3 — Supporting Information 3 Figure S2: Molecular docking of candidate antigens with the human immune receptor TLR4. Representative docking models of human TLR4 in complex with each recombinant antigen are shown: (A) rP1, (B) rP2, (C) rP3, (D) rP4, (E) rP5, and (F) rP6. Dashed boxes indicate the predicted interaction interfaces, which are enlarged in the lower panels; residues involved in the interface are listed on the right. [file TBED-2026-3394193-s003.tif]

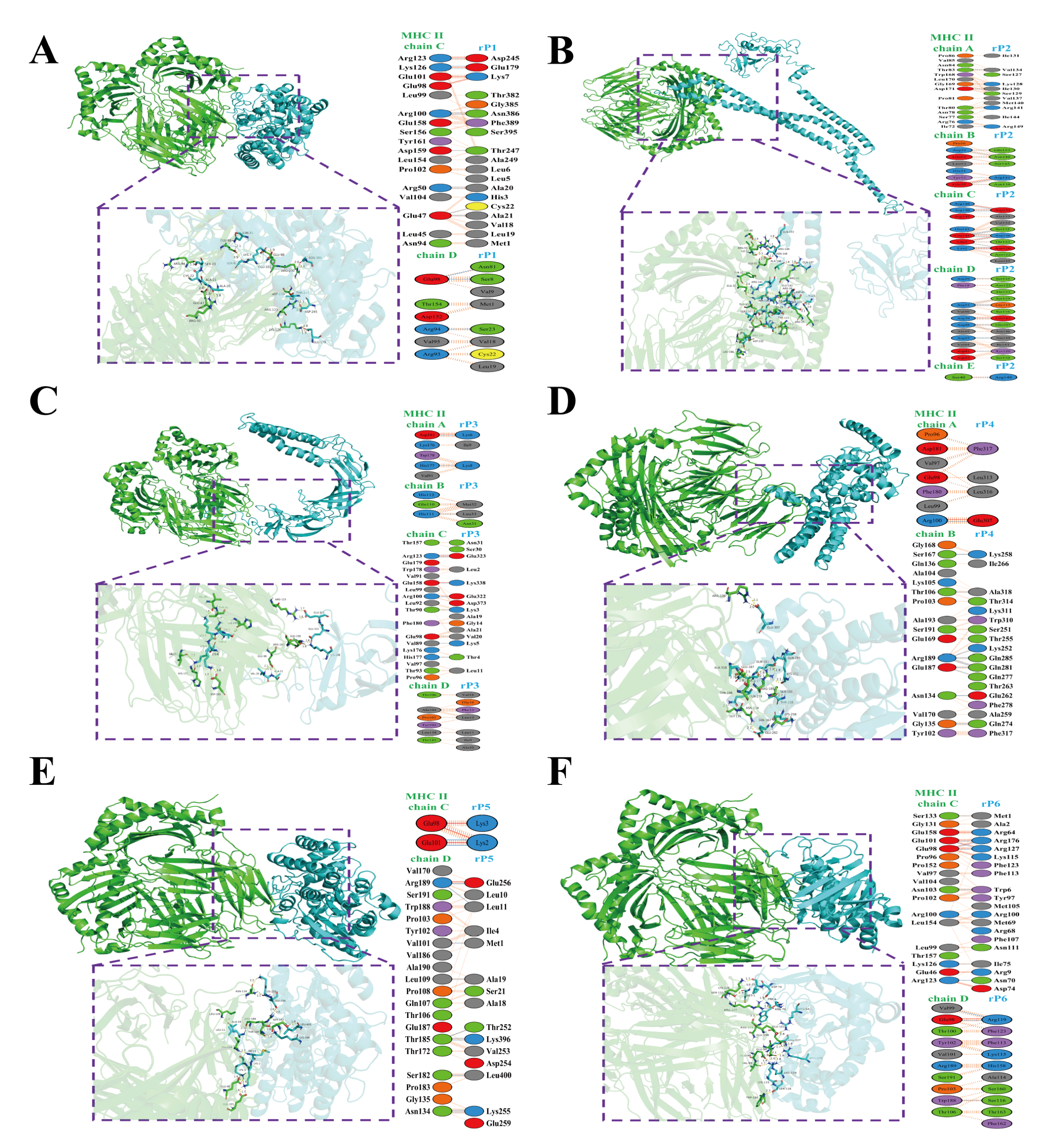

Supplement: Supplementary file 4 — Supporting Information 4 Figure S3: Molecular docking of candidate antigens with the human immune receptor MHC class II. Representative docking models of human MHC II in complex with each recombinant antigen are shown: (A) rP1, (B) rP2, (C) rP3, (D) rP4, (E) rP5, and (F) rP6. Dashed boxes indicate the predicted binding interfaces, which are enlarged in the lower panels; residues involved in the interface are listed on the right. [file TBED-2026-3394193-s002.tif]

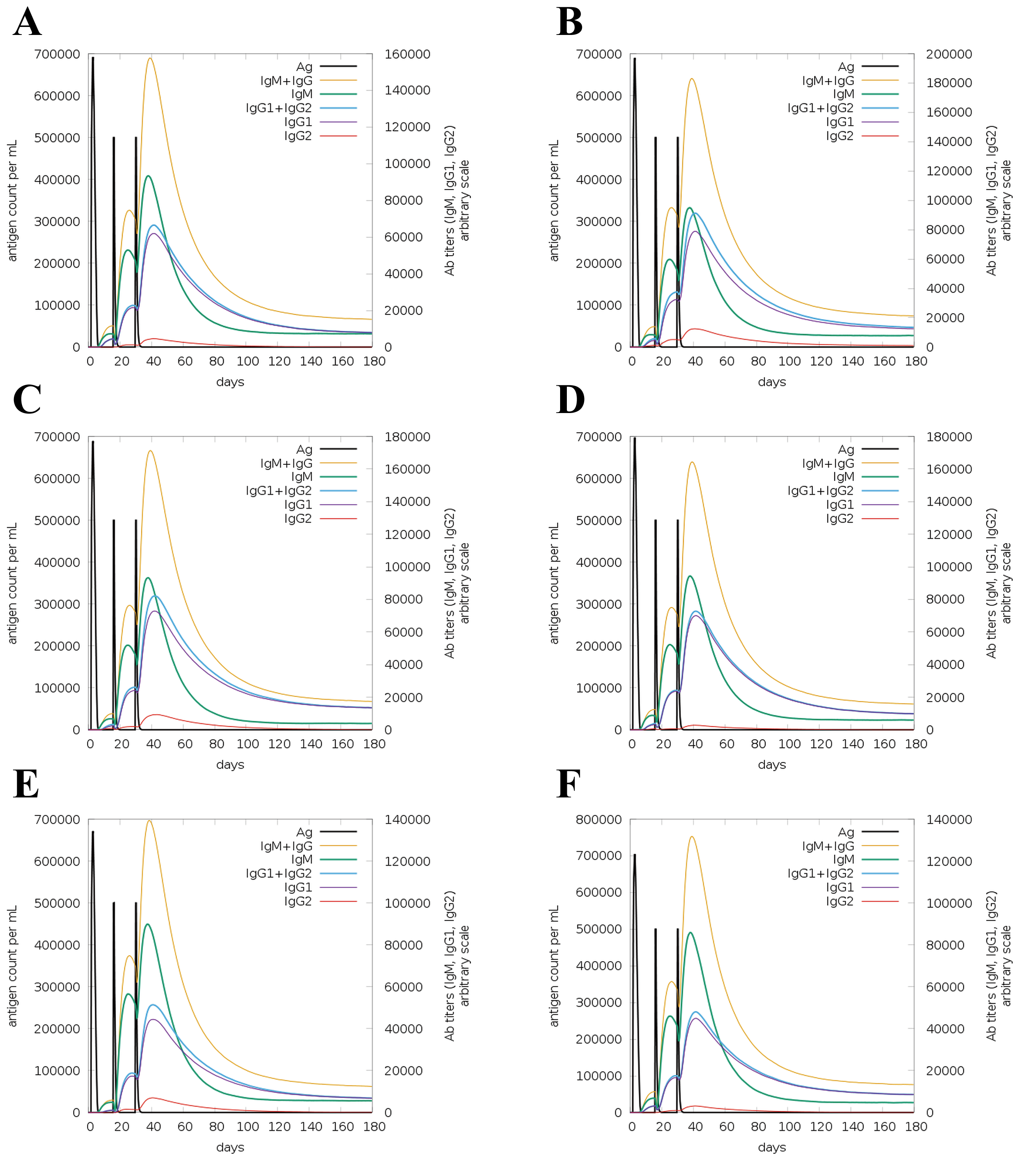

Supplement: Supplementary file 5 — Supporting Information 5 Figure S4: Simulated immune responses induced by candidate antigens. In silico immunization profiles show the predicted kinetics of antigen levels and antibody responses, including IgM, IgG, and IgG subclasses, following repeated antigen exposures for each candidate: (A) rP1, (B) rP2, (C) rP3, (D) rP4, (E) rP5, and (F) rP6. [file TBED-2026-3394193-s001.tif]

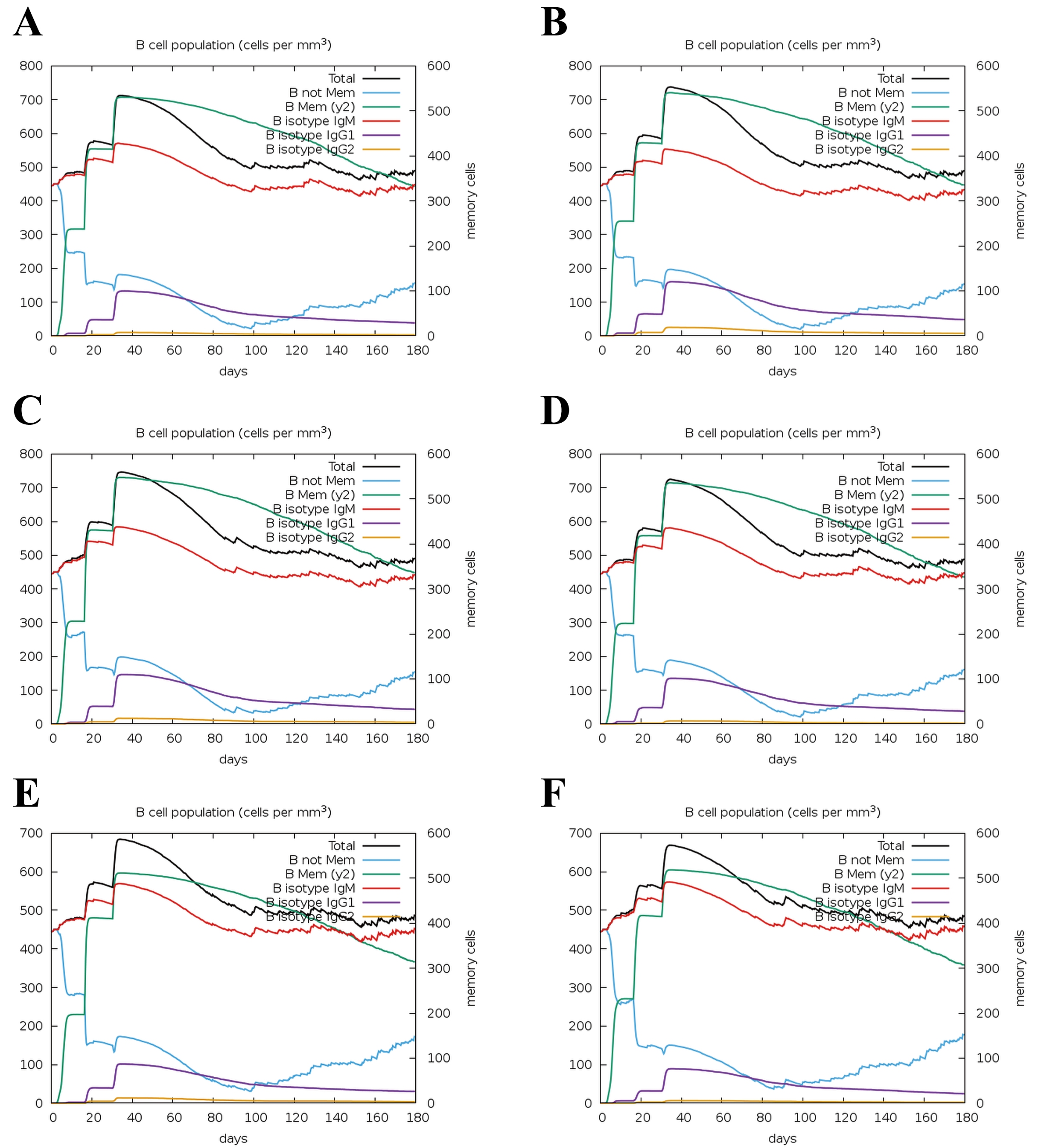

Supplement: Supplementary file 6 — Supporting Information 6 Figure S5: Predicted B‐cell dynamics following simulated immunization with the candidate antigens. In silico immune simulations depict the kinetics of total B cells, nonmemory B cells, memory B cells, and isotype‐specific B‐cell responses over time for each candidate antigen: (A) rP1, (B) rP2, (C) rP3, (D) rP4, (E) rP5, and (F) rP6. [file TBED-2026-3394193-s005.tif]
